# MRS2179: a novel inhibitor of platelet function

**DOI:** 10.1186/1753-6561-9-S1-A2

**Published:** 2015-01-14

**Authors:** H Dunne, J Cowman, D Kenny

**Affiliations:** 1 Cardiovascular Biology, Royal College of Surgeons in Ireland, 123 St. Stephen’s Green, Dublin 2. Ireland

## Background

Antiplatelet agents, such as aspirin and P2Y12 inhibitors, are essential in the secondary prevention of cardiovascular disease [1]. Despite effective treatment with these drugs, many patients still suffer ischemic events. This suggests the need for additional antiplatelet therapy. The P2Y1 receptor is a seven transmembrane G protein coupled receptor responsible for platelet shape change and reversible aggregation [2]. Animal studies have shown that antagonists of the P2Y1 receptor, such as MRS2179, inhibit platelet aggregation [3]. The effect of P2Y1 inhibition in man is not yet clear. To address this we characterised platelet function in human blood using a novel shear-mediated dynamic assay.

## Methods

Blood used was drawn from healthy donors free from antiplatelet medication. Light transmission aggregometry (LTA) was used to determine the optimal concentration of MRS2179. Platelet aggregation was induced by the addition of incremental concentrations of ADP. The optimal concentration of MRS2179 to inhibit ADP induced aggregation was 20µM. Thrombus formation in vivo occurs due to the tethering, adhesion and translocation of platelets to von Willebrand Factor (vWF) under arterial shear conditions [4]. To test the effect of MRS2179 under these conditions blood was perfused at an arterial shear rate of 1500-s through custom made parallel plate flow chambers coated with purified vWF. Platelets were labelled with a fluorescent dye and images were recorded at 30 frames per second. A novel software programme used distance weighting to calculate the amount of static and translocating platelets, the mean distance travelled by the platelets, the translocation velocity, the percentage of platelets moving at one time and the percentage of the surface covered in 500 frames.

## Results

The results of this study demonstrate that a concentration of 20µM of MRS2179 effectively inhibits aggregation. In 13 normal donors 20µM either completely inhibited ADP induced aggregation or enhanced platelet disaggregation (p<0.05). In preliminary experiments from 3 normal donors assayed there were no significant changes in most of the parameters measured in the dynamic assay. However, platelet translocation velocity in the presence of the P2Y1 antagonist was significantly increased (p<0.05).

## Conclusions

Selective inhibition of the P2Y1 surface receptor results in a significant decrease in aggregation in the presence of an agonist. Preliminary data using a novel dynamic assay of platelet function suggests that P2Y1 inhibition may be of therapeutic value.
